# Mental health professionals' perceptions, judgements and decision-making practices regarding the use of electronic cigarettes as a tobacco harm reduction intervention in mental healthcare: A qualitative focus group study

**DOI:** 10.1016/j.abrep.2019.100184

**Published:** 2019-05-02

**Authors:** Charlie Albert Smith, Ann McNeill, Loren Kock, Zoyah Ahmed, Lion Shahab

**Affiliations:** a Department of Behavioural Science and Health, University College London, 1-19 Torrington Place, London WC1E 6BT, UK; b National Addiction Centre, King's College London, 4 Windsor Walk, London SE5 8BB, UK; c UK Centre for Tobacco and Alcohol Studies, UK

**Keywords:** THR, tobacco harm reduction, EC, electronic cigarette, MHP, mental health professional, NICE, National Institute for Health and Care Excellence, RCP, Royal College of Physicians, NHS, National Health Service, Tobacco harm reduction, Electronic cigarettes, *E*-cigarettes, Mental healthcare, Qualitative research

## Abstract

**Background:**

Smoking prevalence remains significantly higher among individuals with mental health conditions compared with the general population. Tobacco harm reduction (THR) in the form of replacing cigarettes for electronic cigarettes (ECs) is an alternative approach which may prove useful for these smokers who find it difficult to quit. Exploring how mental health professionals' (MHPs) perceive ECs, and how these influence decision making regarding their use in clinical settings is essential to determine the feasibility of incorporating ECs into the treatment pathway.

**Methods:**

We conducted six focus groups between March and August 2017. A total of 39 MHPs were recruited from mental healthcare services in England. Discussions were guided by a semi-structured guide, and responses were recorded, transcribed and coded using thematic framework analysis.

**Results:**

MHPs generally adopt a risk-averse approach when judging the safety and suitability of ECs. Risk-aversion was influenced by perceived obscurity surrounding ECs and THR, as well as high exposure to adverse and unreliable information regarding ECs, and perceived analogies between ECs and conventional cigarettes. Some MHPs adopt a pragmatic approach when making decisions based on THR and EC use in daily practice by considering the context of treatment and patient circumstances. However, this is often accompanied by a high degree of caution and misconceptions which limits the potential benefit this intervention could have in mental healthcare settings.

**Conclusion:**

Improved dissemination of national guidance and scientific literature regarding THR and ECs is essential in mental healthcare to eliminate misconceptions and better inform MHPs evidence-based decision-making practices.

## Highlights

• Quitting smoking is difficult to achieve among smokers with mental health conditions.
• ECs may be a promising harm reduction intervention for these smokers.
• MHPs hold misconceptions regarding ECs which may undermine this intervention.
• Improved dissemination of the THR and EC evidence-base is needed in mental healthcare.

## 1 Background

Tobacco control is a key government initiative within the United Kingdom (UK) (Department of health, 2017). In England, smoking prevalence in the general population has declined from 24% in 2007, to 14.9% in 2017 (NHS digital, 2018). However, smoking prevalence among individuals with mental health conditions in England has remained high; estimated at around 34.1% in 2014 (Richardson, McNeill, & Brose, 2019). Consequently, smoking within this population contributes to an average of 17 life years lost (Harker & Cheeseman, 2016). Specifically, in the United States (US), research indicates that smokers with severe psychological distress (SPD) lose 14.9 years of life relative to never-smokers without SPD, and 9.6 years relative to non-smokers with SPD (Tam, Warner, & Meza, 2016). In an attempt to reduce smoking prevalence in disadvantaged populations such as those with mental health conditions, the National Institute for Health and Care Excellence (NICE) in the UK has issued guidance and recommendations to adopt tobacco harm reduction (THR) as an alternative approach to cessation for those who find it difficult to quit (NICE, 2013). THR is a strategy in which smokers are able to substitute cigarettes for less harmful nicotine-containing products, thus essentially maintaining a desired level of nicotine intake while minimising exposure to harmful chemical components present in tobacco smoke (Royal College of Physicians, 2016).

Smoking is a complex bio-psycho-social behaviour incorporating biological dependency, habits, beliefs and identity (Hughes, 2002). Although traditionally licensed harm reduction products such as nicotine replacement therapies (NRT) deliver nicotine to the user and thus satisfy the biological dependency, these products fail to address the behavioural and social aspects of smoking. Subsequently, these products may not be well suited for highly dependent smokers, such as those with mental health conditions (Ratschen, 2014). In contrast, electronic cigarettes (ECs) relieve nicotine cravings, while simultaneously replacing the behavioural and social aspects of smoking (Farrimond, 2017; Wadsworth, Neale, McNeill, & Hitchman, 2016). ECs are able to achieve this as they are non-combustible, non-tobacco products, which typically allow the user to inhale a vaporous solution of nicotine suspended in a humectant (typically propylene glycol and glycerine), thus providing nicotine delivery via a hand-to-mouth movement (McNeill et al., 2018). In the UK, these products have become the most popular smoking cessation aid, with an estimated 3.2 million EC users in 2017 (Action on Smoking and Health, 2018). Furthermore, as more evidence emerges supporting their use for harm reduction purposes, bodies such as Public Health England (PHE) and the Royal College of Physicians (RCP) in the UK, as well as the National Academy of Sciences in the US, have concluded that smokers who are unable to quit should consider converting to vaping as a safer alternative to conventional cigarettes (McNeill et al., 2018; National Academies of Sciences, 2018; Royal College of Physicians, 2016).

In the context of mental healthcare, ECs may be one solution to reduce smoking in these settings by functioning as a safer source of nicotine (Ratschen, 2014). Research from the US indicates that smokers with mental health conditions are more likely to have tried an EC and be a current EC user in comparison with smokers in the general population (Cummins, Zhu, Tedeschi, Gamst, & Myers, 2014). What is more, a small number of randomised controlled trials (RCTs) and pilot studies from Italy (Caponnetto, Auditore, Russo, Cappello, & Polosa, 2013), Australia (O'Brien, Knight-West, Walker, Parag, & Bullen, 2015) and the UK (Hajek et al., 2019; Hickling et al., 2018) have found that ECs are effective in supporting smoking cessation and harm reduction among smokers with mental illnesses. As a result of this growing body of evidence, the Science and Technology Committee in London, England, have advised mental health trusts to allow EC use on their premises (Science and Technology Committee, 2018).

However, research has found that MHPs are often reluctant to support their patients to address their smoking, due to widely held misconceptions regarding the necessity and appropriateness of smoking cessation interventions in mental healthcare (Sheals, Tombor, McNeill, & Shahab, 2016). Specifically, MHPs hold intrinsic biases regarding patients' abilities and motivation to quit, and believe quitting smoking is too much for patients to take on (Sheals et al., 2016). Nevertheless, it remains unclear as to whether MHPs would be willing to support ECs and THR as an alternative approach to cessation. Research to date has only explored the acceptability of ECs among general healthcare personnel, with findings showing a degree of support from some (Kandra, Ranney, Lee, & Goldstein, 2014; Lazuras, Muzi, Grano, & Lucidi, 2016), and lack of support from others (Hiscock et al., 2014; Van Gucht & Baeyens, 2016). In any instance, misperceptions and concerns are prominent among healthcare personnel (Marques Gomes, Nabhani-Gebara, Kayyali, Buonocore, & Calabrese, 2016; Pippard & Shipley, 2017; Stepney, Aveyard, & Begh, 2019), though there is some research to suggest that there is high demand among healthcare personnel to know more about ECs in order to better inform decisions regarding their use by patients (Stepney et al., 2019). However, no research exists exploring this among those working specifically with smokers who have mental health conditions. This is of particular interest given that smokers with mental health conditions are likely to turn to their healthcare providers for advice and information regarding these products.

In light of the NICE guidance on THR and recommendations from the Science and Technology Committee regarding the use of ECs in mental health settings, the present study aims to qualitatively explore how MHPs perceive THR and ECs, and and how these influence their decisions regarding their use in clinical settings.

## 2 Methods

### 2.1 Sample and recruitment

To recruit a diverse participant group to resemble the multidisciplinary nature of mental healthcare teams, a stratified purposive sampling method was adopted. The lead author (CS) achieved this by using the NHS England website to search for appropriate mental health services local to the author that treated a diverse range of mental health conditions. CS contacted appropriate services via email, and those who indicated interest and capacity to participate were forwarded further information about the purpose of the study.

In total, 39 MHPs participated in the present study; 36 were recruited from five services within one of the largest mental health trusts in Europe, and 3 were recruited from one service within another mental health trust in London (See Table 1 for a breakdown of participant and service characteristics). Services specialised in treatment for a range of mental health conditions, including common mental disorders (e.g. anxiety and depression), mood disorders, psychosis and personality disorders.

**Table 1:** Participant and service characteristics.

Participant characteristics (*n* = 39)	Mean (SD)
Age (years)	37.4 (13.5)^a^
Years in current role	6.4 (7.8)^a^
Gender	*n* (%)
Male	11 (28.2)
Female	28 (71.8)
Service type	*n* (%)
Secondary care inpatient wards	1 (16.7)
Secondary care community services	3 (50)
Primary care community services	2 (33.3)
Participants in each service	*n* (%)
Secondary care inpatient wards	4 (10.3)
Secondary care community services	15 (38.5)
Primary care community services	20 (51.3)
Professional discipline	n (%)
Occupational Therapist^b^	1 (2.6)
Psychiatrist	4 (10.3)
Clinical Psychologist^b^	4 (10.3)
Trainee Clinical Psychologist	2 (5.1)
Health Psychologist	1 (2.6)
Assistant Psychologist	1 (2.6)
Psychological Wellbeing Practitioner (PWP)	8 (20.5)
Trainee PWP	2 (5.1)
IAPTS placement student	1 (2.6)
Cognitive Behavioural Therapy (SBT) Therapist	1 (2.6)
Nurse^b^	5 (12.9)
Student Nurse	1 (2.6)
Social worker	4 (10.3)
Forensic Mental Health Practitioner	1 (2.6)
Support worker	1 (2.6)
Administrator/assistant	2 (5.1)
Self-reported smoking status	*n* (%)
Non-Smoker	18 (46.2)
Ex-Smoker	14 (35.9)
Occasional/social smoker	6 (15.4)
Smoker	1 (2.6)

a Age was not disclosed by 6 MHPs.

b Includes acting service leaders (one occupational therapist, one clinical psychologist, and three nurses).

### 2.2 Data collection

A total of six focus groups (one for each participating service) were conducted between March and August 2017, with each lasting 45–60 minutes. The decision to conduct focus groups over individual interviews was made following two pilot interviews and one pilot focus group, where it was decided that focus groups allowed for more broadening exploration and opportunity for MHPs to generate and share ideas/concerns regarding ECs and harm reduction. The rationale for conducting focus groups for each service individually was that participants would be able to engage in honest and elaborative discussion regarding ECs and THR with known colleagues, thus theoretically increasing the real-life applicability of this study's findings by providing insight into the decision-making processes of multidisciplinary teams.

The focus groups were facilitated by CS, with the assistance of ZA. All focus groups began with everyone introducing themselves to the group. CS and ZA explained the purpose of the focus group, which was read from the topic guide (Appendix A). Participants were provided with the opportunity to ask questions after the information sheet was provided and read aloud by CS or ZA, and participants were asked to provide written consent before the focus groups began. All focus groups were conducted in an interview room on the hospital site where the services were based.

The facilitators used a semi-structured interview guide to direct the discussions and prompt MHPs when needed. This guide was informed following a review of the literature and was refined using an iterative process following piloting in two interviews and one focus group before the study began. The final topic guide (Appendix A) covered numerous topics relating to tobacco control interventions and mental health:

1. Experiences of providing patients with smoking cessation support
2. Attitudes/perceptions toward THR
3. Attitudes/perceptions toward ECs
4. Attitudes/compliance of smoke-free policy in mental healthcare

For the purpose of the aims of this research paper, the data obtained from questions three and four (regarding THR and ECs) were included in the analysis of this paper. Data regarding MHPs' experiences of providing smoking cessation interventions and attitudes/compliance toward the smoke-free policy are reported in another qualitative paper by the same authors (Smith, McNeill, Kock, & Shahab, 2019).

### 2.3 Ethical considerations and review

Participants received an information sheet at the start of each focus group. This information was read aloud by either CS or ZA at the start of each focus group. Participants were advised orally of their rights and were assured of anonymity before they provided written consent. The project was reviewed and approved by the University College London ethics committee (application reference 10093/001).

### 2.4 Data analysis

With participants' permission, focus groups were audio recorded and transcribed verbatim by CS, and were checked for accuracy by ZA. Transcripts were interpreted and analysed using thematic analysis with the framework method (Gale, Heath, Cameron, Rashid, & Redwood, 2013). This approach was chosen as framework analysis consists of clearly defined steps to follow and produces highly structured outputs of summarised data. Data was managed using Nvivo (version 11) software (Bazerley & Jackson, 2013) where authors (CS, LK and ZA) recorded initial descriptive themes. These authors independently developed over-arching themes and sub-themes, before reviewing each other's draft themes and developing an agreed interpretation with the input from fellow authors (AM and LS). CS charted the data into a framework matrix, which involved summarizing the data for each theme and case. Finally, a developed interpretive framework was produced by CS, and the remaining authors reviewed and agreed on the final framework.

## 3 Results

### 3.1 Theme 1: MHPs adopt a risk-averse approach when forming perceptions and judgements regarding ECs safety and suitability

#### 3.1.1 Sub-theme 1: perceived obscurity surrounding THR and ECs facilitate misconceptions among MHPs

MHPs generally indicated a lack of awareness and knowledge of ECs and THR. MHPs that had more knowledge about ECs, and the impact of tobacco use on the mental health population, supported the use of ECs in mental healthcare. However, many MHPs were unaware of the literature available on ECs. Uncertainty regarding the contents of ECs led some MHPs to be cautious in their judgements by questioning whether ECs are indeed a ‘safer’ alternative to smoking:

“It's difficult to know as well how bad e-cigarettes are. I don't think there is much research or general information about what are the dangers, because they are a relatively new thing. So I guess having a safe alternative; what does that actually mean? What is a safe alternative? Are e-cigarettes a safe alternative?”

(**Robbie, Community Setting, PWP, Non-Smoker**)

In many cases, MHPs indicated their personal preferences for traditional NRT over ECs, in spite of recognising patients are generally not interested in these products. However, this was often in the context of cessation. Many MHPs were cautious to recommend ECs over NRT, as ECs were perceived less as an intervention to smoking and more as a potential lifestyle:

“I think with NRT there is that expectation that you are going to reduce down, so it's clearer. But with vaping, maybe not so much, and the paraphernalia around it, there is more potential for it to be a lifestyle rather than a stepping stone towards complete cessation.”

(**Margaret, Community Setting, Clinical Psychologist, Non-Smoker**)

This uncertainty surrounding the benefits of THR and ECs often perpetuated MHPs beliefs that ECs are products which prolong nicotine addiction, rather than support patients to overcome an addiction. This continued use of nicotine was perceived by many to be problematic, which impaired MHPs ability to recognise the benefit of ECs, and in some cases NRT use, over combustible tobacco use:

“There is a plastic thing, a small one. I don't think I've ever seen someone suck on anything as hard on that than the patients on the ward when they were given that instead of access to cigarettes. It almost seemed their nicotine intake was more than if they just had the odd fag during the day because they were constantly 24 hours a day you would see them, then they would go and get a refill. Really sucking really deeply… I don't know what it does or what's in it, but it just struck me… I couldn't really see what the point of it was other than stopping them for actually inhaling smoke.”

(**Nigel, Community Setting, Clinical Psychologist, Ex-Smoker**)

[…]

“The more it goes on the more upset I am about prescribing all these extras because sometimes people use more nicotine than if they were allowed to smoke every two hours.”

(**Kelly, Community Setting, Psychiatrist, Ex-Smoker)**

#### 3.1.2 Sub-theme 2: increased engagement with unreliable information sources over trust resources perpetuate misconceptions among MHPs

The majority of MHPs were unaware of their trust's smoke-free policy with regard to EC use, although many MHPs spoke about completing an annual online smoking cessation training module which forms part of their mandatory professional development. However, only a minority of MHPs recalled content regarding EC use, and only one MHP indicated that they had read scientific literature supporting EC use in comparison with cigarettes:

“I think there has been recent literature published that suggests the harms from e-cigarettes in their current commercialised state are less than the harms from cigarettes.”

(**Sharon, Community Service, Psychiatrist, Ex-Smoker**)

In contrast, the majority of MHPs were unable to recall EC content covered on the training module which they had completed in the past year, and in one case, the day before the focus group. Yet, many recalled extreme cases of where they had heard or read ECs to be dangerous or destabilising to wider tobacco control policy:

“I can't remember if ECs are covered in the e-learning… I can't remember if I read or heard it, but it was over the weekend, that young people are taking up vaping rather than smoking, which that's not what its meant to be… its meant to be an aid to stopping, isn't it.”

(**Hannah, Community Service, Nurse & Team Leader, Social Smoker**)

[…]

“I read something in the paper that the percentage of harm they are doing compared to cigarettes is not a lot of percentage difference.”

(**Charlotte, Inpatient Service, Admin and ward assistant, Non-Smoker**)

[…]

“When I did my e-learning yesterday they had all these awful chemicals in a cigarette; arsenic and you mentioned tar, and toxins really that are in the cigarette. So is that not in the e-cigarette?”

(**Debbie, Inpatient Service, Nurse and Manager, Ex-Smoker)**

In rare instances, this was taken further by some MHPs who stated that any nicotine use poses threat to users' health:

“Well look at the spray, do you remember the spray? I swear they said it was causing tongue cancer or throat cancer. So I think if you want to stop smoking you should just stop altogether… I don't think NHS should be prescribing nicotine for people at all.”

(**Mandy, Community Service, Social Worker, Ex-Smoker**)

#### 3.1.3 Sub-theme 3: analogies between ECs and conventional cigarettes triggers suspicion among MHPs when making judgements regarding THR and ECs

Much criticism directed toward ECs appeared to be somewhat driven by the perceived similarities between ECs and cigarettes; including visual stimuli and behavioural actions produced during the use of both products. This appeared to lead many MHPs to mistakenly consider ECs to be a “type” of conventional cigarette, whereby the same guidelines and terminology would apply to these users. These MHPs therefore did not see the benefit of substituting a cigarette for an EC:

“I think sometimes if you want a cigarette you kind of want the real thing. I don't know how helpful ECs would be because it would still be the same rules that you would have to leave and smoke, you couldn't smoke on the wards, and with e-cigarettes it's the behaviour you have to look at.”

(**Charlie, Community Setting, Mental Health Practitioner, Social Smoker**)

Even among MHPs who had used ECs and thus supported their use for THR, the perceived similarities shared between smoking and vaping influenced the language which was used when discussing past experiences of using these products. One occasional smoker who considered himself an “ex-vaper” believed ECs to be better than NRT in the sense that ECs provide a similar experience to using cigarettes. However, this MHP explained how he perceived EC users to remain classified as smokers, which was the consensus shared by many MHPs:

“If you just want to do harm reduction and you don't want to really stop smoking then I think its fine to use an electronic cigarette because your lungs definitely feel better on an e-cigarette… I couldn't do research in it, but I think it just sounds logical that it's safer than smoking cigarettes. The problem is then you're stuck on that so what's the point? You're still an addict to nicotine… you're not classified as a non-smoker”.

(**Martin, Community Setting, Psychiatrist, Social Smoker**)

Many MHPs spoke about ECs as being the new craze in the smoking world; referencing the increase in number of vape shops on the highstreets over recent years. Discussions regarding the perceived glamorisation of ECs, along with product advancement and diversity, led one MHP to express how he felt this to be a marketing strategy employed by the tobacco industry to maximise profit that was being threatened due to the decrease in tobacco smoking prevalence in the general population:

“Well cynically I feel that the tobacco industry who own all this as I understand as well, so to think people aren't going to smoke cigarettes that they are making sure that they corner the market to make e-cigarettes as elaborate as they can to make sure that people spend a lot of money on them”

(**Jack, Community Setting, Social Worker, Ex-Smoker**)

The perceived similarity between cigarettes and ECs led many MHPs to predict that ECs would be exposed in the future as being harmful products; replicating the timeline of events with regard to cigarettes which were once endorsed by medical professionals, but were later exposed as lethal products:

“I think ideally if someone wants to stop smoking then they should stop smoking altogether. I think replacing it for lesser evil… in terms of harm, in five or ten years' time we might be given new information that it causes physical health problems as well.”

(**Jenny, Community Setting, Nurse, Social Smoker**)

### 3.2 Theme 2: MHPs consider patient circumstances when making judgements regarding EC use for THR purposes in clinical practice

#### 3.2.1 Sub-theme 1: treatment context

Judgements regarding the suitability of using ECs for THR within a clinical setting were sometimes dependent on the treatment context and patients' individual circumstances. For instance, one MHPs expressed how decisions regarding whether or not to allow patients' to use ECs, or even conventional cigarettes, depended upon the patients' stage of treatment. This was spoken in the context of working with patients who have a history of substance abuse:

“I've seen people really suffer and struggle because they are not just doing the patches, they are doing the chewing gum, and they are doing everything! And they are hiding it, and it becomes a new… it's almost like hiding a bottle of gin, but you're not, you're hiding your patches and tablets. So I think it depends on the individual and obviously it's going to depend on if people are in recovery… it depends on what levels people are at. Obviously if people are really unwell, then in the grand scheme of things, it's not the end of the world, because like you said, life is so difficult, it's so hard, so let me have that vape or cigarette. Let's be real. But, when things do start to improve, with some people with that history of any difficulties with substances, it's going to be quite different, the process of trying to stop.”

(**Judith, Community Setting, PWP, Ex-Smoker**)

Some MHPs expressed how ECs may prove to be particularly advantageous by providing a similar experience to smoking for inpatients that are unable to smoke outside of hospital grounds due to their confinement on the wards. Others elaborated on ECs' potential in providing comfort to these patients who smoke partly for the behavioural rituals:

“What comes to mind is that there are some wards where I know people can't maybe step out or have leave to smoke… so maybe having an alternative like an e-cigarette in that situation maybe something that can help them in that situation.”

(**William, Community Setting, PWP, Current Smoker**)

A minority of MHPs also proposed that ECs could be a positive alternative for patients who experience anxiety and thus use the rituals of smoking as a coping mechanism for symptom management. This was discussed in the context of traditional NRT not being able to provide these cues:

“So if you think about patients who suffer from anxiety, every time they feel quite anxious they go out for a cigarette. It might be that actual physical thing of holding the cigarette in their hand and going outside that the vape would replace, rather than just sitting there with the patch and feeling quite anxious and not using that behaviour.”

(**Patricia, Community Setting, Trainee PWP, Non-Smoker**)

#### 3.2.2 Sub-theme 2: patient-centred focus

Patient choice was taken into consideration by some MHPs when determining whether ECs would be a helpful THR product for patients within their care. One MHP reflected on the high smoking prevalence in this population, which was further used to justify the use of ECs in a mental healthcare context:

“I think it just needs to be absolutely individualised and led by patient choice. The patient absolutely needs to be in control of the plan because they are going to have to live the plan.”

(**Sharon, Community Service, Psychiatrist, Ex-Smoker)**

[…]

“When you first asked the question, I thought wow that's really stigmatising, why should we treat people with severe mental illness differently from people in the general population, why shouldn't we give them the same care? But I think the key is that prevalence is different, so peer group is different, so pressure to smoke is different. So, abstinence might not be a feasible goal, which makes harm reduction more, you know…”

(**Sharon, Community Service, Psychiatrist, Ex-Smoker)**

Even among MHPs who were sceptical of ECs, a minority spoke of accounts whereby they had encouraged EC use by patients. Although such accounts were rare and predominantly in the context of inpatient settings, some MHPs expressed how they would encourage patients who are already using ECs to take them on escorted leave, in attempt to dissuade them from purchasing tobacco which would inflict financial strain on the patient.

“It's a good way to encourage them as well before we go out on leave, because it's so expensive so I'm like why don't you just take your pipe out so you can at least save that £4 and spend it on shopping instead of spending it on tobacco? It's such a waste of money now.”

(**Charlotte, Inpatient Service, Admin and ward assistant, Non-Smoker)**

Finally, one MHP spoke about how, in spite previously raising concerns regarding ECs, she allows ECs to be used by patients in their rooms. Moreover, this MHP elaborated on how, in spite of being ill-informed regarding ECs, she had read the smoke-free policy and believed they serve to be a popular alternative source of nicotine for patients who could not smoke:

“The rule is that they are meant to do it in the bedroom area and we have to constantly encourage them to do that, because what you don't want to do is normalize any form of smoking behaviour.”

(**Debbie, Inpatient Service, Nurse and Manager, Ex-Smoker**)

[…]

“What I felt was I didn't want the patients to suffer, I didn't want them to be unhappy, I felt that saying “you can't smoke and that's it, I don't want NRT doctor thank you very much”, that it was an alternative to support them. And I still maintain that view… but I do wonder what is in them [ECs].”

(**Debbie, Inpatient Service, Nurse and Manager, Ex-Smoker**)

## 4 Discussion

To our knowledge, this is the first study to explore MHPs perceptions of THR and ECs, as well as the decision-making processes underlying their use in mental health clinical settings. MHPs adopt a risk-averse approach when making judgements regarding the safety and potential effectiveness of ECs in mental healthcare. Specifically in the context of the present study, this was predominantly influenced by a perceived obscurity surrounding ECs and THR, high prevalence of and reliance upon unscientific and misleading information, and perceived analogies between ECs and conventional cigarettes. Among MHPs who expressed a degree of endorsement for ECs, this was almost exclusively on a patient by patient basis rather than expressing general support for these products. Specifically, patients' diagnosis and treatment setting were considered, along with other individual circumstances.

We report elsewhere that MHPs in the current study experience constraints in their capability, opportunity and motivation to address smoking with their patients, including having the belief that smoking is not a priority behaviour to address with mental health patients (Smith et al., 2019). This is a persistent issue which has not only been found in mental healthcare, but also among healthcare professionals who work with other socially disadvantaged groups experiencing high smoking prevalence, including those in addiction services and the homeless (Cookson et al., 2014; Garner & Ratschen, 2013). Interestingly, these socially disadvantaged smokers are often just as motivated to quit compared to the general population, and interest in EC and experimentation are common among these smokers (Dawkins et al., 2019; Hefner, Valentine, & Sofuoglu, 2017; Spears et al., 2018). However, smoking prevalence remains disproportionately higher in these groups, suggesting that these smokers are lacking the support required to convert exclusively to ECs. In relation to the present study, MHPs hold many misconceptions and unfounded concerns regarding ECs which may be preventing smokers to convert to ECs within these services. Therefore, it is crucial for research to explore the cause of these concerns, in order to develop interventions to minimise them. One explanation may be that MHPs heavily rely on mental shortcuts when making decisions regarding ECs, especially in light of resource constraints (Smith et al., 2019). For instance, judging ECs based on perceived analogies with conventional cigarettes may be explained by the cognitive bias known as representative heuristics, and the tendency to recall information frequently presented by the media over less frequent training content and scientific publications may be the consequence of information and availability bias (Tversky & Kahneman, 1974) (Table 2 presents finding from theme 1 using a cognitive psychology framework). Indeed, it is widely known that negative press regarding ECs tends to be more prevalent in the media compared with positive messages (McNeill et al., 2018), and so it may not necessarily be of surprise that such sources are being utilised, particularly among MHPs who feel they lack resources to address smoking with patients (Smith et al., 2019).

**Table 2:** A cognitive psychology framework of the present findings (with exemplar quotes).

Potential cognitive biases	Example quotes	Participant characteristics
Ambiguity effect/risk aversion	“I think with NRT there is that expectation that you are going to reduce down, so it's clearer. But with vaping, maybe not so much, and the paraphernalia around it, there is more potential for it to be a lifestyle rather than a stepping stone toward complete cessation.”	Clinical psychologist, non- smoker
	“I wonder how healthier the e-cigs are anyway? I haven't read up too much about them, but they don't seem that healthier because it has got the chemicals”	Healthcare assistant, social smoker
Representativeness heuristic	“I think sometimes if you want a cigarette you kind of want the real thing. I don't know how helpful ECs would be because it would still be the same rules that you would have to leave and smoke, you couldn't smoke on the wards, and with e-cigarettes it's the behaviour you have to look at.”	Mental health practitioner, social smoker
	“There is something different they put in [ECs] that's bad for you. If you look at it this way, from say 10 years ago, where can you smoke now? Nowhere. So that would make people cut down naturally because you can't smoke anywhere. But e-cigs you can smoke anywhere.”	Admin and ward assistant, non-smoker
Focalism	“There is a plastic thing, a small one. I don't think I've ever seen someone suck on anything as hard on that than the patients on the ward when they were given that instead of access to cigarettes. It almost seemed their nicotine intake was more than if they just had the odd fag during the day because they were constantly 24 h a day you would see them, then they would go and get a refill. Really sucking really deeply… I don't know what it does or what's in it, but it just struck me… I couldn't really see what the point of it was other than stopping them for actually inhaling smoke.”	Clinical psychologist, ex-smoker
	“The thing is with smoking it's trying to get them away from their addiction, and that is not getting them away from their addiction. Even though it's better for their health it's still an addiction. They are still addicted to something.”	Admin and ward assistant, non-smoker
Availability heuristic	“I can't remember if ECs are covered in the e-learning… I can't remember if I read or heard it, but it was over the weekend, that young people are taking up vaping rather than smoking, which that's not what its meant to be… its meant to be an aid to stopping, isn't it.”	Nurse & team leader, social smoker
	“I read something in the paper that the percentage of harm they are doing compared to cigarettes is not a lot of percentage difference.”	Admin and ward assistant, non-smoker

### 4.1 Future research

In light of the present findings, more efforts are needed to improve the knowledge about ECs among MHPs, in order to guide clinical practice and decision making with respect to ECs as a THR intervention. Due to the heterogeneity of the services involved in the present study, future work should explore attitudes and decision-making regarding THR and ECs in each area of mental health (i.e. inpatient, community, primary care, etc.) to generate more in-depth issues that may be unique to different service types. Moreover, future research should expand this to other settings supporting vulnerable groups presenting with high smoking prevalence and low cessation rates, including those who present with poor MH and coexisting health and social needs (e.g. homelessness and substance users).

### 4.2 Strengths and limitations

The evidence-base around ECs is constantly developing and so the findings are limited to the time when they were gathered and analysed. These qualitative data from a small purposive sample cannot be generalized to the population of mental health professionals in the UK or internationally, and so further research is warranted. Focus groups were used to encourage discussions that were interactive thus generating further data; however, this means it is not possible to eliminate the possibility of a ‘bandwagon effect’, whereby participants agree with the view of others against their own internal beliefs. This may be particularly the case in focus groups where MHPs had differing opinions to those in positions of authority (i.e. team leaders or managers). However, the fact that focus groups involved professionals with backgrounds from a wide variety of disciplines also serves as a strength to this study by representing views of a multidisciplinary culture which is often the case in healthcare. This is valuable, as studies in the past have often solely recruited medical professionals, such as general practitioners and nurses. Finally, as is true of qualitative research in general, our account cannot claim to provide a fully objective view, given intrinsic biases and influences of the researchers involved in the analysis. However, we did attempt to triangulate findings by deviant case analysis and discussion about and agreement on themes among co-authors.

## 5 Conclusion

In summary, our findings provide insight into how MHPs perceive THR and ECs, and how they make decisions regarding the implication of such approaches in mental healthcare. These findings suggest that improved dissemination of the evidence-base supporting THR and ECs as effective tobacco control interventions is warranted. Further exploration is required in order to determine how the acceptability of THR and ECs can be improved among MHPs, as this is essential if such approach is to be successfully integrated across the mental healthcare pathway as a viable tobacco control approach for smokers with mental health conditions.

## Conflict of interests

LS is a HEFCE funded member of staff at University College London. He has received honoraria for talks, an unrestricted research grant and travel expenses to attend meetings and workshops from Pfizer and an honorarium to sit on advisory panel from Johnson&Johnson, both pharmaceutical companies that make smoking cessation products. He has acted as paid reviewer for grant awarding bodies and as a paid consultant for health care companies. Other research has been funded by the government, a community-interested company (National Centre for Smoking Cessation) and charitable sources. He has never received personal fees or research funding of any kind from alcohol, electronic cigarette or tobacco companies. All other authors declare no that they have no conflicts of interest.

## Appendix A Supplementary data

Supplementary material Image 1
