# Supplementary material for: Mental health professionals' perceptions, judgements and decision-making practices regarding the use of electronic cigarettes as a tobacco harm reduction intervention in mental healthcare: A qualitative focus group study
Source: Addict Behav Rep. 2019 May 2;10:100184. doi: 10.1016/j.abrep.2019.100184 (PMC6545441; doi:10.1016/j.abrep.2019.100184)
Supplement: Supplementary file 1 — Supplementary material [file mmc1.docx]

| **Scene-setting and instructions to participants (10 mins)** | |
| --- | --- |
| **Introductions**   - *Facilitator and assistant facilitator introduce selves*   **“**Thank you for volunteering to take part in this focus group. Your point of view is important and we appreciate the time that you have given to take part.  This focus group discussion is being held to find out about your thoughts and feelings about smoking amongst people with mental illness and ways to reduce the rate of smoking amongst this group. The discussion should take no more than 45 minutes. As we mentioned in the information about this study, we would like to record this discussion so that we have a record of everything that has been discussed. We would like to assure you that this discussion is confidential and all personal information will be anonymised. The tapes will be kept safely in a locked room until they are transcribed, after which they will be destroyed.  Can I just confirm that everyone is happy for the discussion to be recorded?  We’d like to encourage you to answer and comment as accurately and truthfully as possible. To protect everyone’s confidentiality, we would also like to ask you to refrain from discussing the comments of other group members outside of the focus group. Whilst we’d like you to be as involved as possible, if there are any questions or discussions that you do not want to answer to of participate in, you do not have to do so. If at any point you’d like to withdraw from the study, you are free to leave at any point.”  **Cover ground rules:**   - Important to treat focus group as a relaxed discussion - it is not a question and answer session. - Only one person speaking at a time - please wait until others have finished before commenting. - No right or wrong answers - we are just interested in what you have to say. - Please say what you really think and feel, and feel free to respond to other people’s comments. There’s no need to wait for me to ask you questions. - Any questions before we begin? | |
| **Introductions (5 mins)** | |
| - *Ask all group members to introduce themselves by name and role, and state what they had for breakfast (ice-breaker). Ask participants to speak clearly to facilitate identification of individuals during transcription.* | |
| **Main discussion (45 – 60 mins)** | |
| ***Questions*** | ***Prompts*** |
| 1. To start this discussion off, what are your experiences of offering patients advice or assistance to quit smoking, and how could you have overcome any difficulty in doing this? | *Have these experiences been positive or negative?*  *Do you feel it is part of your job role? If not, why not?*  *Do you think it has/would have any effects on your relationship with patients?*  *-Any experiences of it doing so?*  *-Any cases where it has helped?* |
| 1. What do you think about the idea of harm reduction (i.e. replacing some or all cigarette smoking with a less harmful product)? | *Do you think it would be an effective or appropriate approach?*  *Do you think it has any benefits over complete cessation?*  *Can you foresee any problems with taking this approach?* |
| 1. What do you think about electronic cigarettes? | *Do you feel you have enough knowledge of e-cigarettes as a harm reduction tool?*  *Any benefits or drawbacks in comparison to other treatments?*  *Any benefits or drawbacks in people with mental health issues?*  *How much do you know about the different types of e-cigarettes?*  *Any personal experiences of using them?* |
| 1. How would you feel about offering e-cigarettes to patients to help them to stop or reduce smoking? | *What are your experiences of demand for e-cigarettes from service users?*  *What would make this difficult to do?*  *What would make this easier to do?* |
| 1. The recent Smoke Free Policy includes guidance on facilitating service users’ use of e-cigarettes. Have you read the policy? What are your thoughts on this policy? | *Do you have any experiences of implementing this policy?*  *Have you had any difficulties in implementing this policy?*  *Do you have any suggestions for how the policy could be improved?* |
| *****FOR FORENSIC TEAMS*****   1. Do you think that working with forensic service users has or would have any effect on how you do or would address their smoking behaviours? |  |
| 1. Before we finish this discussion, is there anything else anyone would like to add? *(ask each group member individually)* |  |
| **Debrief and end** | |
| “Thank you to everyone for coming along – we’ve had a really productive and interesting discussion and you’ve all made some great contributions.”  *(Give everyone an opportunity to request results of the study and provide feedback)* | |
